# Drugs of Abuse and Their Impact on Viral Pathogenesis

**DOI:** 10.3390/v13122387

**Published:** 2021-11-29

**Authors:** Jason T. Blackard, Kenneth E. Sherman

**Affiliations:** 1 Division of Digestive Diseases, Department of Internal Medicine, University of Cincinnati College of Medicine, Cincinnati, OH 45267-0595, USA; 2 Center for Addiction Research, University of Cincinnati College of Medicine, Cincinnati, OH 45267-0595, USA

**Keywords:** drug use, opioid, HIV, hepatitis B virus, hepatitis C virus

## Abstract

Commonly misused substances such as alcohol, cocaine, heroin, methamphetamine, and opioids suppress immune responses and may impact viral pathogenesis. In recent years, illicit use of opioids has fueled outbreaks of several viral pathogens, including the human immunodeficiency virus (HIV), hepatitis B virus (HBV), and hepatitis C virus (HCV). This review focuses on the myriad of mechanisms by which drugs of abuse impact viral replication and disease progression. Virus–drug interactions can accelerate viral disease progression and lead to increased risk of virus transmission.

## 1. Drug Use Morbidity and Mortality in the US

There were over 19 million people aged 18 or older with substance use disorder (SUD) in the United States in 2019 [1]. Frequently misused substances include alcohol, cocaine, heroin, marijuana (cannabis), tobacco, methamphetamine, and opioids. Given the sharp rise in opioid-related deaths in recent years, this review focuses on opioids and their interactions with common viral pathogens, such as the human immunodeficiency virus (HIV), hepatitis B virus (HBV), and hepatitis C virus (HCV).

Rates of drug overdose deaths have increased significantly in recent years [2,3]. From 2016 to 2017, more than 142,000 Emergency Department visits were suspected opioid-involved overdoses [4]. Among the 70,000+ drug overdose deaths in 2017, 67.8% involved an opioid [5]. In 2019, 70.6% of drug overdose deaths involved opioids, while 51.5% involved synthetic opioids [6]. Contributing to these increased deaths is the dramatic increase in the availability of heroin and illicitly manufactured fentanyl and fentanyl analogs [7,8,9,10,11,12].

## 2. Drug Use and Immune Function

Several commonly misused substances suppress immune responses (reviewed in [13,14]). Possible mechanisms include impaired function of natural killer cells, T cells, B cells, neutrophils, dendritic cells, and/or macrophages, altered expression of cytokines and chemokines, and the weakened integrity of the intestinal barrier, all of which contribute to decreased ability to control pathogens and limit their subsequent clearance. Evidence of the immunosuppressive effects of drug use are further supported by epidemiologic studies demonstrating an increased rate of infections amongst persons with SUD [15,16,17]. 

## 3. Common Viral Infections Associated with Drug Use

Drug use—particularly the opioid pandemic—has fueled outbreaks of several viral pathogens. Perhaps the most well-known example occurred in Scott County, Indiana, in 2015. In an area that had previously recorded approximately five new HIV cases annually, over 200 people were diagnosed with HIV in less than one year [18,19]. Most individuals lived in rural communities, were under 40 years of age, white, and nearly half were women. A total of 80% reported injection drug use. Among those, all reported dissolving and injecting oxymorphone tablets. It was subsequently reported that most individuals were also co-infected with HCV [20]. Increases in HIV and/or viral hepatitis associated with injection drug use have been noted in several other settings [21,22,23,24,25,26,27]. A meta-analysis found that 17.8% of persons who inject drugs (PWIDs) were living with HIV, 52.3% were HCV seropositive, and 9.1% were HBV surface antigen positive [28]. Thus, it is not surprising that rises in viral infections associated with injection drug use—frequently involving opioids—have been noted internationally as well [29,30,31,32,33,34].

## 4. Opioids

There are three main types of opioids. Natural opiates are found in plants and include morphine and codeine. Semi-synthetic opioids are those created in laboratory settings from natural opiates and include hydromorphone, hydrocodone, and oxycodone, as well as heroin, which is made from morphine. Synthetic opioids include fentanyl, fentanyl analogs, buprenorphine, methadone, and tramadol. Endogenous opioid peptides are also responsible for a plethora of physiological functions and include endorphins, enkephalins, and dynorphins.

Opioid family receptors are classified as μ-opioid (MOR), δ-opioid (DOR), κ-opioid (KOR), and nociceptin/orphanin (ORL) receptors [35]. When an opioid receptor is activated, adenylyl cyclase is inhibited, leading to the activation of K^+^ channels and the reduced conductance of Ca^2+^ channels. Opioid receptors also activate mitogen-activated protein kinases and phospholipase C-mediated signaling, leading to the formation of IP3 and diacyl glycerol. The activation or inhibition of downstream signaling cascades thus facilitates the intrinsic effects of opioids [36,37].

## 5. Opioids and HIV

**Figure 1:**
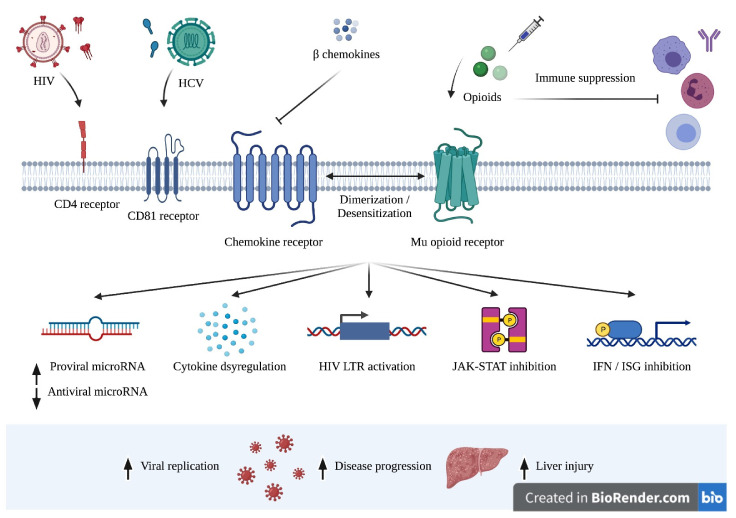
Mechanisms of virus–drug interactions that lead to increased viral replication and accelerated disease progression for HIV and HCV, and/or liver injury.

Opioid receptors are expressed on a variety of immune cells, such as lymphocytes, macrophages, neutrophils, and monocytes [38,39]. Therefore, the interactions between opioids/opioid receptors and HIV are important to investigate (Figure 1). Endogenous opioid peptides enhance HIV expression. For instance, the β-endorphin enhanced viral protein production and long terminal repeat (LTR) activation in microglia [40]. Similarly, endomorphin-1 increased HIV expression in mixed glial/neuronal, as well as microglial, cell cultures [41], and dynorphin upregulated HIV expression in fetal brain co-cultures [42]. However, subsequent studies have not evaluated the role of endogenous opioid peptides on HIV disease progression in vivo.

Squinto et al. first reported that morphine activated the HIV LTR in human neuroblastoma cells [43]. Subsequent studies showed increased HIV replication with several cell types. Morphine increased HIV expression in promonocyte/fetal brain cell co-cultures, as well as primary cultures of Kupffer cells [44,45]. Morphine also triggered viral reactivation in latently-infected lymphocytes [46]. Interestingly, Wang et al. demonstrated that morphine withdrawal also enhanced HIV replication within peripheral blood lymphocytes, as well as T cell lines in vitro [47]. The neuropeptide substance P (SP) enhanced viral replication in a dose-dependent manner, and an SP antagonist inhibited the effect of morphine withdrawal on HIV replication. HIV LTR activation was also increased in cells undergoing morphine withdrawal. The same group reported that morphine upregulated chemokine receptor expression, downregulated β chemokine production, inhibited the expression of interferons (IFNs), IFN-inducible genes, and regulators of the Janus Kinase signal transducer and activator of transcription (JAK–STAT) signaling pathway, and decreased expression of anti-HIV microRNAs, while also increasing HIV expression in blood monocyte-derived macrophages and neonatal macrophages [48,49,50,51]. Morphine withdrawal also enhanced HIV expression within macrophages and inhibited expression of multiple viral inhibitory factors [52]. Additionally, Balinang et al. also demonstrated increased HIV replication in neural progenitor cells in the presence of morphine [53]. Notably, HIV proteins also synergize with morphine to increase the expression of opioid receptors, alter cell cycle regulation, and exacerbate neurotoxicity and neuroinflammation [54,55,56,57,58,59,60].

Using an in vitro HIV-CD4+ T cell system, Liang et al. showed that morphine treatment induced more drug-resistant mutations under selective pressure from antiretroviral drugs and shortened the generation time for such mutations compared to controls treated with only antiretroviral drugs [61]. The inhibitory effects of antiretroviral therapies on HIV replication in primary astrocytes in vitro were also attenuated by morphine [62]. Morphine influences antiretroviral drug concentration in a drug and cell type-dependent manner [57], although the in vivo significance of these findings remains to be determined. In the simian immunodeficiency virus (SIV) model, morphine alters the fecal microbiome and expression of microbial metabolites and reduces the CD4+ T cell reservoir in lymphoid tissues, while increasing the microglia/macrophage reservoir in the central nervous system [63,64]. There are no published reports on whether another natural opioid—codeine—has similar effects on HIV infection/replication.

Of the semi-synthetic opioids, data on their impact on HIV pathogenesis are only available for heroin (heroin triggered HIV reactivation in latently-infected lymphocytes in vitro) [46]. Others have demonstrated that heroin enhanced HIV expression in macrophages, suppressed IFNs, and inhibited several anti-HIV microRNAs [51,65]. Altered microRNA expression was also confirmed in peripheral blood mononuclear cells (PBMCs) and/or macrophages from heroin-dependent persons [65,66]. In a large cohort study, HIV-infected PWIDs had higher plasma HIV RNA levels than non-PWIDs, and PWIDs expressed lower levels of host restriction factors such as TRIM5α, TRIM22, ABOBEC3G, and IFNs than individuals with no drug use [67].

Methadone and buprenorphine are widely used for the treatment of opioid addiction. Patient-based studies investigating their impact on HIV pathogenesis are lacking. However, in vitro studies indicated that methadone enhanced HIV replication within fetal microglia and blood monocyte-derived macrophages, and increased replication in latently-infected PBMCs [68]. Methadone increased expression of CCR5 in monocyte-derived macrophages. A subsequent study by the same group found that methadone also increased HIV expression in primary macrophages and reduced expression of IFNs, IFN-stimulated genes, and several anti-HIV microRNAs [69].

Retention on buprenorphine treatment was associated with maximal HIV suppression in opioid-dependent persons [70], although there are no reports on whether buprenorphine directly impacts HIV levels in vivo. A recent study found that buprenorphine resulted in more than an eight-fold increase of in vitro infection of PBMCs from uninfected individuals with an HIV reporter virus [71]. In contrast, in a murine model of HIV, viral levels were not increased, although cognitive impairment was reduced in the presence of buprenorphine [72].

Despite the significant contribution of synthetic opioids such as fentanyl to the current opioid crisis, data evaluating the potential association of fentanyl or fentanyl analogs on HIV disease are limited. We recently reported preliminary data suggesting that fentanyl increases HIV replication via enhanced expression of the CCR5 and CXCR4 chemokine co-receptors in several cell types [73,74]. Nonetheless, the impacts of other synthetic opioids on HIV pathogenesis remain to be explored.

## 6. Opioid Receptor/Chemokine Receptor Interactions

It is well established that, like chemokines, μ-, δ-, and κ-opioids induce chemotactic responses in monocytes and neutrophils [75,76,77,78]. It has also been shown that the activation of mu- and delta-opioid receptors leads to the heterologous desensitization of chemokine receptors (reviewed in [79]). Thus, opioid receptor–chemokine receptor interactions and desensitization by opioid agonists may have important implications for HIV pathogenesis. Several groups have sought to antagonize opioid receptor–chemokine receptor interactions as a novel therapeutic strategy to limit HIV replication. Akgün et al. synthesized a bivalent MOR agonist/CCR5 antagonist that exhibited significant anti-nociception in mice [80]. This bivalent compound was around 3500 times more potent than a mixture of the MOR agonist and CCR5 antagonist monovalent ligands. Arnatt et al. developed a different bivalent compound targeting the MOR–CCR5 heterodimer [81]. Exposure to the bivalent ligand significantly reduced HIV p24 levels in PBMCs, macrophages, and primary astrocytes. This group subsequently developed a bivalent MOR–CXCR4 antagonist that is around 150 times more potent than monovalent controls at inhibiting HIV entry [82], and have recently reported additional bivalent MOR–CCR5 ligands with potent anti-HIV activity [83].

## 7. Stimulants and HIV

Stimulants such as cocaine and methamphetamine may alter HIV pathogenesis and have been reviewed in detail elsewhere [84,85]. For instance, stimulant use is associated with higher HIV RNA levels and greater CD4+ T cell decline [86,87,88]. In vitro, cocaine enhances HIV transcription through the altered expression of NFκB, mitogen- and stress-activated kinase 1 (MSK1) p38 mitogen-activated protein kinase (p38 MAPK), and/or initiation and elongation factors [89,90,91]. Cocaine can directly enhance HIV expression in multiple cell types including PBMCs, macrophages, CD4+ T cells, dendritic cells, microglia, and astrocytes [89,92,93,94,95,96,97,98,99]. Peterson et al. further reported that antibodies to either tumor necrosis factor alpha (TNFα) or transforming growth factor beta (TGFβ) reduced the impact of cocaine on HIV replication [94]. Cocaine can also enhance HIV neuroinvasion by remodeling microvascular endothelial cells in the brain [100]. Roth et al. noted a 100- to 300-fold increase in HIV levels after cocaine administration in a murine model of HIV infection [101,102]. The mechanism by which cocaine influences HIV pathogenesis includes increased chemokine co-receptor expression, epigenetic modifications, upregulation of dendritic cell-specific intercellular adhesion molecule-3-grabbing non-integrin (DC-SIGN), differential MAPK expression, dysregulation of arachidonic acid and its metabolites, and/or downregulation of anti-HIV microRNAs [90,91,93,98,99,102,103,104,105].

Methamphetamine use in the US increased 43% from 2015 to 2019 [106]. Methamphetamine use alters the immune system in a variety of ways, including altered immune cell subset number, increased pro-inflammatory cytokine production, increased CD4+ and CD8+ T cell proliferation, enhanced CD4+ T cell activation and/or exhaustion, and altered immune-related signaling pathways, as reviewed elsewhere [107,108,109,110]. Population-based studies report that methamphetamine use is associated with higher HIV viral loads, higher likelihood of having detectable HIV viral loads, and/or lower CD4+ T cell counts compared to non-users [111,112,113]. Several in vitro studies have shown that methamphetamine and the HIV tat protein interact to alter mitochondrial dysfunction and cell death [114,115]. Others have demonstrated increased HIV replication in the presence of methamphetamine in multiple cell types, including blood monocyte-derived macrophages, monocyte-derived dendritic cells, neural progenitor cells, and CD4+ T cells [116,117,118,119,120,121,122,123,124]. The proviral effects of methamphetamine on HIV are attributed to the enhanced expression of chemokine co-receptors, decreased expression of β chemokines, inhibition of IFNα, upregulation of T cell activation markers, altered microRNA expression, dysregulation of signal transduction pathways, inhibition of TLR9, and activation of the HIV LTR. 

## 8. Alcohol and HIV

Alcohol represents a near ubiquitous extrinsic factor that can play a role in infection, transmission, and maintenance of chronic viral infections. The World Health Organization reports that alcohol consumption contributes to 3 million deaths per year globally (WHO 2018). The 2019 National Survey on Drug Use and Health reported 139.7 million current alcohol users aged 12 and older in 2019 [125].

Alcohol can impact HIV by increased risk of infection/transmission, reduced viral suppression, and increased emergence of viral resistance to antiretroviral therapies, as well as alcohol-associated immunosuppression that increases the risk of opportunistic infections and disease progression. A meta-analysis of 10 studies reported that any alcohol consumption was associated with an increased relative risk of HIV infection, while binge drinking led to an increased relative risk of 2.2 [126]. This risk is attributable in part to the link between alcohol use, unprotected sexual intercourse, multiple sexual partners, and a prior history of sexually transmitted infections [127,128]. Recreational drug use, intimate partner violence, and depression are also recognized as co-morbidities of alcohol misuse [129].

Suppression of HIV with antiretroviral therapy has implications for the infected individual in terms of disease progression, and as well as HIV transmission to others. In a longitudinal study of patients in continuity care, the time spent with a viral load >1500 copies/mL was evaluated. Hazardous alcohol use, recent drug use, black race, and age were highly associated with failure to maintain an HIV <1500 copies/mL [130]. In the Women’s Interagency HIV Study, heavy drinking was associated with failure to remain virally suppressed as well [26]. The association between failure to suppress HIV and disease progression due to alcohol is well established. However, the factors that contribute to disease progression are less clear. A detailed review of this subject suggests that alcohol may affect HIV progression through alteration of the microbiome, affecting gut permeability, and systemic activation, though not all studies report an association between heavy alcohol use and markers of progression [131]. Alcohol may increase risk of infections in persons with HIV by altered immune surveillance mechanisms. Experimental models suggest that alcohol consumption increases susceptibility to pneumococcal pneumonia [132]. The presence of alcohol use disorder is associated with greater pneumonia severity as well [133]. In vitro studies suggest that HIV gp120 and alcohol increase blood-brain barrier permeability [134]. Additionally, others have demonstrated increased HIV replication in the presence of alcohol in various cell types, including T lymphocytes, monocyte-derived macrophages, monocyte-derived dendritic cells, epithelial cells, and oral keratinocytes [47,135,136,137,138,139,140]. Higher alcohol-induced levels of HIV may lead to increased risk of transmission to others. The mechanisms by which alcohol enhances HIV replication include upregulation of chemokine co-receptors and inhibition of β chemokines [137].

## 9. Opioids and Viral Hepatitis

Opioid receptors are expressed in the liver where they are important mediators of liver disease progression [141,142,143,144,145,146,147]. Hepatic stellate cells (HSCs) are major contributors to liver fibrosis and express multiple opioid receptors [141,142,144,145,146,148]. However, less is known about the impact of opioids on HCV and liver disease (Figure 1). In vitro studies demonstrated that morphine, heroin, and methamphetamine enhance HCV replication [149,150,151,152,153]. We recently reported that fentanyl increased replication of HCV and HBV in hepatocytes [73]. The addition of fentanyl also resulted in significant apoptosis. RNA sequencing identified multiple hepatocyte genes that were differentially regulated by fentanyl, including those related to apoptosis, the antiviral interferon response, chemokine signaling, and NFκB signaling. As higher virus levels are associated with pathogenesis and virus transmission, additional research is essential to our understanding of opioid-virus pathogenesis and for the development of new and optimized treatment strategies. However, there are no data on HCV RNA levels and/or markers of liver damage in fentanyl-using populations. Similarly, there are no published reports evaluating HCV or HBV attachment or entry factors by fentanyl or other opioids. While multiple microRNAs stimulate or suppress HCV replication [154], studies of opioid HCV–miRNA interactions are absent from the literature.

## 10. Stimulants and Viral Hepatitis

As with HIV, stimulants such as cocaine and methamphetamine may alter the pathogenesis of viral hepatitis. However, data regarding the influence of cocaine on viral hepatitis in at-risk populations are sparse. While cocaine use is associated with HCV infection and the presence of HCV viremia, the impact of cocaine on HCV RNA levels or treatment response rates is less clear [155,156,157]. There are conflicting data on the impact of cocaine use on liver disease [158,159]. Similarly, there are limited studies on the impact of cocaine use on HBV-related liver disease, although amongst US veterans with HCV, cocaine and other drug use was associated with HBV co-infection [160].

Ye et al. demonstrated that methamphetamine increased HCV replication and inhibited hepatic IFNα expression in vitro [149]. Population-based studies evaluating the impact of methamphetamine on HCV RNA levels and liver disease are limited. However, methamphetamine injection is an independent predictor of incident HCV infection [161,162,163], and recent methamphetamine injection is associated with phylogenetic clustering of HCV, suggesting that it plays a role in HCV transmission networks [164].

## 11. Alcohol and Viral Hepatitis

Alcohol and viral hepatitis (HBV or HCV) are important contributors to liver injury and progression to cirrhosis, leading to increased morbidity and mortality. The interaction between HCV and alcohol misuse is significant and appears to be dose- and duration-dependent. For example, an analysis of National Health and Nutrition Examination Survey (NHANES) data revealed that excessive alcohol consumption was associated with a hazard ratio for liver-related mortality of around 184 compared to around 74 for HCV-infected persons not reporting excessive alcohol consumption. Liver-related mortality was increased with moderate alcohol use, as well as when HCV was present [165]. Among those cured of HCV with directing acting agents, unhealthy alcohol use remained associated with liver-related outcomes and mortality, although low-level alcohol use was not [166]. A myriad of factors could influence liver injury and disease progression among HCV-infected persons who drink alcohol. Several studies have suggested an increased quasispecies complexity of HCV associated with alcohol use [167,168]. In vitro studies have described increased viral replication following the exposure of permissive cells to alcohol [169]. It has been reported that upregulation of the microRNA-122 that facilitates HCV replication could be involved [170]. Alcohol is also associated with HCV replication in PBMCs [171]. Liver injury is also associated with the development of steatosis and free radical oxidative stress, both of which are increased in conjunction with HCV and alcohol exposure [172].

The impact of alcohol on HBV is poorly understood. Yet, alcohol consumption increases HBV surface antigen (HBsAg) and viral DNA in transgenic mice [173], supporting a previous study demonstrating increased HBsAg levels in HepG2 cells in the presence of ethanol [174]. Nonetheless, cohort-based studies evaluating the impact of alcohol on HBV-specific liver disease are lacking.

## 12. Tobacco, Cannabis, and Viral Infections

Among persons with HIV, the prevalence of cigarette smoking is estimated at 40% to 70% (reviewed in [175,176]). Multiple studies have reported that cigarette smoke exposure is associated with increased HIV replication, lower CD4+ T cell counts, immune activation, oxidative stress, and/or decreased adherence or response to antiretroviral therapy (ART) [177,178,179]. Smoking/nicotine also enhances HIV replication in macrophages, T lymphocytes, and/or microglia [180,181,182,183,184]. Ranjit et al. observed a three- to four-fold increase in HIV replication in macrophages exposed to benzo(a)pyrene—a major carcinogen found in cigarettes. The impact of cannabis use on HIV is less clear with some studies reporting a reduced adherence to ART and missed clinical appointments, while others reported beneficial/no impact on virus levels [185,186,187,188,189]. Notably, given its potential anti-inflammatory properties, cannabis has been offered as a potential treatment for addiction, as well as for neuroinflammation associated with HIV [190,191].

Recent reviews show cannabis use may impact liver disease [192]. While some studies have reported that cannabis use was associated with liver fibrosis, other studies showed little to no effect. Cannabis use may also be associated with reduced hepatic steatosis [193].

## 13. Drugs of Abuse and SARS-CoV-2

The ongoing SARS-CoV-2/COVID-19 pandemic has raised considerable concern about its potential negative impact on persons with SUD [194,195]. Social isolation and stress increased with lockdowns during the pandemic. Addiction services and treatment options were also severely limited to align with new social distancing norms. Recent reports now confirm that drug overdose deaths increased during the COVID-19 pandemic [196,197,198,199]. Wang et al. recently reported that persons with SUD were at significantly increased risk for COVID-19 and worse outcomes [200]. Fentanyl use also increased during the COVID-19 pandemic [201,202]. COVID-19 mortality may be exacerbated in persons with SUD for several reasons, including downregulation of interferon expression, development of pulmonary edema, increased thrombotic factors, and increased expression of angiotensin-converting enzyme 2 (ACE2) (reviewed in [203], although data to support these potential mechanistic interactions are quite limited currently). However, the acute nature of infection with SARS-CoV-2 may prove challenging for evaluating how drugs of abuse impact SARS-CoV-2/COVID-19 pathogenesis in vivo.

## 14. Recommendations for Additional Research

There are multiple future research opportunities to consider related to drugs of abuse and chronic viral infections. Firstly, side-by-side comparison of multiple drugs of abuse and their impact on viral replication in vitro is essential using standardized drug doses, established timings of drug exposure, and cell types that are relevant to the viral life cycle and the overall disease progress in vivo. Secondly, polysubstance abuse is quite common in persons with substance use disorders. While this clearly complicates study design and data analysis, the impact of polysubstance abuse compared to single drugs of abuse is not commonly considered in well-characterized clinical cohorts with viral infections and co-infections. Thirdly, the opioid antagonists naloxone and naltrexone can limit the increased viral replication brought about by distinct drugs of abuse in vitro [48,50,65,153,204,205,206,207,208]. Furthermore, a recent clinical trial demonstrated that extended-release naltrexone improves HIV viral suppression, thus suggesting its potential role in the treatment of SUD in persons with chronic viral infections [209]. Similarly, high rates of HCV clearance were achieved with combined direct-acting antivirals and opioid agonist therapy in HCV-positive PWIDs [210]. Thus, increased availability and utilization of opioid antagonists may have beneficial impacts on the most vulnerable and at-risk populations. Nonetheless, the potential effects of these agents on synthetic opioid-mediated virus replication have not been evaluated to date in vitro or in at-risk patient populations. Fourthly, given their central role in HIV pathogenesis, as well as liver disease, and the possibility of opioid receptor–chemokine receptor interactions, several groups have sought to antagonize these interactions as a novel therapeutic strategy to limit HIV replication. Yet, this strategy could be extended to include liver disease caused by alcohol, HBV, and/or HCV. A more robust understanding of the complex interactions between opioids, viruses, and the chemokine system could facilitate the optimization of therapeutic options for patients with SUD and lead the to the development of novel therapeutic strategies for multiple diseases that are common in PWIDs. Fifthly, the interactions of drugs of abuse with antiretroviral therapies for HIV or direct-acting agents for HCV are largely known, as reviewed elsewhere (reviewed in [211,212,213]). However, progress towards the development of new antiviral drugs, as well as new drug formulations, and the creation of new, distinct drugs of abuse, such as fentanyl analogs, requires careful study. Moreover, the impact of viral infections on drug–drug interactions and drug metabolism must be considered. Sixthly, the human T-lymphotropic virus type 1 (HTLV-1) which infects around 10 million people globally can lead to adult T-cell leukemia/lymphoma and HTLV-associated myelopathy/tropical spastic paraparesis (reviewed in [214]). Given its routes of transmission, co-infections with HIV, HBV, and HCV are relatively common and would likely impact persons with SUD/OUD (opioid use disorder). However, there are almost no published data on the impact of drugs of abuse on HTLV-1 pathogenesis. Thus, this represents an excellent focus for future research.

## 15. Conclusions

Chronic viral infections like HIV, hepatitis B, and hepatitis C have a significant impact on individuals, and the health of societies as a whole. Though suppressive or curative strategies are available for these infectious diseases, their efficacy and impact may be limited by the use or misuse of drugs and/or alcohol through a variety of interconnected mechanisms. These include effects on viral replication, immune-mediated clearance, and medication adherence. The outcomes of these interactions can lead to disease progression (i.e., CD4+ T cell decline, immune activation, and/or increased hepatic fibrosis/cirrhosis) and to increased risk of transmission. While virus–drug interactions have been explored for some drugs of abuse, there are limited data for others. Additional research into the complex interactions between drugs of abuse and viral pathogenesis must simultaneously occur at the level of basic research in vitro, as well as in vivo in large cohort studies that are sufficiently powered to permit the analysis of subsets of individuals experiencing these disease modifiers.
